# Standard Terminologies for Photoplethysmogram Signals

**DOI:** 10.2174/157340312803217184

**Published:** 2012-08

**Authors:** Mohamed Elgendi

**Affiliations:** 1 School of Engineering and Information Technology, Charles Darwin University, Australia; 2 School of Electrical and Electronic Engineering, Nanyang Technological University, Singapore; 3 Institute of Media Innovation, Nanyang Technological University, Singapore;; Affiliated with Royal Darwin Hospital, Darwin, Australia

**Keywords:** Plethysmogram, acceleration photoplethysmogram, second derivative photoplethysmogram, digital volume pulse, pulse wave, pulse oximeter.

## Abstract

Photoplethysmography is one of the optical techniques has been developed for experimental use in vascular disease. It has several advantages over other traditional experimental approaches. Because of its non-invasive, safe, costeffective and easy-to-use properties, it is considered as a useful diagnostic tool. The further developments in the Photoplethysmograph may replace it among other tools used in the assessment of vascular diseases such as blood test and ultrasound. This overview discusses the different terminologies used for the photoplethysmograph and reveals the research discontinuity among different disciplines. Moreover, it suggests standard terminologies as a resolution for a confusion persisted for more than 50 years.

## INTRODUCTION

Photoplethysmography reflects the blood movement in the vessel, which goes from the heart to the fingertips through the blood vessels in a wave-like motion [1], as shown in Fig. (**1a**). It is an optical measurement technique uses an invisible infrared light send in the tissue and the amount of the backscattered light corresponds with the variation of the blood volume [2].

The photoplethysmography (PPG), introduced by Hertzman in 1938 [3], is mainly reflects the blood volume changes in the finger arterioles. It has been recognized as non-invasive, simple, reliable, and cost-effective method of measuring arterial pulse waves in relation to changes in wave amplitude. Therefore, it is considered as a fast developing tool which easily can replace other traditional tools used in the vascular disease assessment such blood test, ultrasound, Doppler, Magnetic resonance angiography and computed tomography angioraphy. However, the wave contour itself has not been analyzed because of the difficulty in detecting minute changes in the phase of the inflections.

In 1978, Ozawa reported (in Japanese) [4] that the second derivative of the photoplethysmogram wave has characteristic contours (waves) that facilitated the interpretation of the original photoplethysmogram waves, as shown in Fig. (**1b**). Therefore, the second derivative of the photoplethysmogram was developed as a method allowing more accurate recognition of the inflection points and easier interpretation of the original plethysmogram wave.

The original photoplethysmogram wave form consists of two waves: systolic and diastolic waves, as shown Fig. (**1a**). The second derivative of the original photoplethysmogram waveform includes four systolic waves and one diastolic wave, namely ***a***-wave (early systolic positive wave), ***b***-wave (early systolic negative wave), ***c***-wave (late systolic reincreasing wave), ***d***-wave (late systolic redecreasing wave) and ***e***-wave (early diastolic positive wave), as shown Fig. (**1b**). 

Although the usage of photoplethysmography has been increasing in science and engineering during the past 50 years, there is still a lack of common terminologies for photoplethysmograph and its second derivative signals. This has caused substantial confusion and disagreement during discussions in literature and in interpretation of the large number of publications in both area science and engineering. This may waste the researcher’s efforts by generating redundant results and also may limit the scientific progress in this multidisciplinary field.

This overview discusses the different plethysmography terminologies and proposes common terminologies to resolve the difficulties and confusion between scientific communities.

## METHODOLOGY

### Photoplethysmogram 

Some of the literature review to explain the different usage of different terminologies for photoplethysmogram:

#### Digital Velocity Pulse (DVP)

In 2002, Millasseau* et al.* [6] used the DVP terminology when they provided a simple, reproducible, non-invasive measure of large artery stiffness using the contour analysis of the DVP.

In 2006, Padilla* et al.* [7] used the DVP terminology when they suggested a useful relation among the stiffness index SI_DVP_ = *h* / ΔT_DVP_ obtainedobtained of simple form by DVP and the ankle-brachial pulse wave velocity (abPWV), as well as the values of sanguineous pressure, whereas abPWV = Lab / ΔT , and Lab measured distance between ankle-brachial location. 

In 2007, Alty* et al.* [8] used the DVP terminology when they developed a method to accurately classify subjects into high and low PWV (equivalent to high and low CVD risk) using features extracted from their DVP waveform has been presented.

#### Plethysmogram (PTG)

In 1999, Mishima *et al.* [9] used the PTG terminology they found the increase in mean RR interval and the decrease in baseline deflection of the PTG strongly correlates of autogenic training.

In 2005, Yashima *et al.* [10] used the PTG terminology when they proposed a new stress evaluation technique using the photoplethysmogram (PTG) by applying Morlet wavelets.

In 2007, Kageyama *et al.* [11] used the PTG terminology when they performed wavelet analysis of the finger-tip photo-plethysmogram (PTG) in order to quantify the stress stage.

#### Photoplethysmogram (PPG)

In 2008, Abe *et al.* [12] used the PPG terminology when they proposed a method for evaluating effects of visually-induced motion sickness using ICA for Photoplethysmography (PPG).

In 2008, Cox *et al.* [13] used the PPG terminology when they proposed the use of photoplethysmogram (PPG) morphology as an indicator of hypovolemic states and its correlation with blood pressure.

In 2009, Gil *et al.* [14] used the PPG terminology when they analysed the heart rate variability (HRV) during decreases in the amplitude fluctuations of photopletysmography (PPG) events for obstructive sleep apnea syndrome screening.

### Second Derivative Photoplethysmogram 

Some of the literature review to explain the different usage of different terminologies for the second derivative plethysmogram:

#### Second Derivative Photoplethysmogram (SDPTG)

In 1998, Takazawa *et al*. [4] used the SDPTG terminology when they demonstrated that the *b/a *ratio reflects increased arterial stiffness, hence the *b*/*a *ratio increases with age. Moreover, they found the *(b-c-d-e)/a* ratio may be useful for evaluation of vascular aging and for screening of arteriosclerotic disease. The waves *a*, *b*, *c*, *d*, and *e* are demonstrated in Fig. (**1b**).

In 1998, Imanaga *et al*. [15] used the SDPTG terminology when they provided a direct evidence that magnitude of *b/a* of the APG is related to the distensibility of the peripheral artery, and suggest that magnitude of *b/a* is a useful non-invasive index of atherosclerosis and altered arterial distensibility.

In 2007, Baek *et al.* [16] used the SDPTG terminology when they confirmed that the *b/a* ratio and the aging index (SDPTG-AI) increased with age, and the *c/a*, *d/a*, and *e/a* ratios decreased with age. The informal aging index *(b-e)/a* has been suggested as a replacement for the known formula *(b-c-d-e)/a*.

In 2007, Kimura *et al*. [17] used the SDPTG terminology when they proposed a the efficacy of Kamishoyosan herb for patients with Premenstrual syndrome was quantitatively ascertained using the APG.

#### Acceleration Photoplethysmogram (APG)

In 1994, Katsuki *et al.* [18] used the APG terminology when they suggested that the present APG index is adequate about the individual reproducibility, and it has the possibility to apply as an index of arteriosclerosis.

In 1996, Takada *et al*. [19] used the APG terminology when they concluded that simply categorized wave patterns of APG could be a useful noninvasive tool to evaluate aging in cardiovascular system.

In 2000, Bortolotto [20] used the APG terminology when they introduced the APG Age Index as a useful measure for evaluation of vascular aging in hypertensives. 

In 2005,Ushiroyama *et al.* [21] used the APG terminology when they reported that patients with a sensation of coldness showed an improvement of the APG index *(b - c - d)/a* upon Sho treatment, which includes Kamishoyosan. Sho is one of the main concepts of Kampo medicine, and corresponds to the holistic tailored treatment suitable for the individual patient’s symptoms. 

In 2006, Nousou* et al. *[22] used the APG terminology when they developed a diagnosis assistance system with a light load to the testee. This system measures the acceleration plethysmogram and reproduces the diagnosis by using Self-Organizing Maps. In 2007, Taniguchi* et al. *[23] used the APG terminology when they proposed a method for using the APG variability to evaluate the surgeon's stress in his/her using a surgical assistant system. In 2008, Fujimoto* et al. *[24] used the APG terminology when they proposed a possibility to diagnosis of stress by accelerated plethysmogram applied the criterion which combines two evaluation based on chaos theory; trajectory parallel measure method and size of neighbourhood space in chaos attractor. 

#### Second Derivative Digital Velocity Pulse (SDDVP)

To date, two papers have been found using the SDDVP. The first by Millasseau* et al.* in 2003 [25] when they used the photoplethysmogram to examine the vascular impact of aging and vasoactive drugs.

The second paper by Rivas-Vilchis* et al.* in 2007, [26] when they used the photoplethysmogram to assess the vascular effects of PC6 (Neiguan) in healthy and hypertensive subjects.

In 2007, Taniguchi* et al. *[23] used the APG terminology when they proposed a method for using the 

 Several general preferences regarding standard terminologies for Photoplethysmogram signals have been introduced as following:

1. Standardization of Photoplethysmogram terminologies is highly desirable.
2. Each terminology system should be as simple as possible and has been used the most in literature.
3. The symbols should indicate the nature of the Photoplethysmogram signal type.
4. A symbol representing a given charge should not be easily confused with any of those representing the other biomedical signals types.

Various sets of symbols were proposed and problems associated with each set were noted as following:

1. Photoplethysmograph, Photoplethysmogram,and Digital Pulse Volume are the same terminologies with different a symbol PTG, PPG, and DVP.
2. Second Derivative Photoplethysmograph, Acceleration Photoplethysmograph are the same terminologies with different symbols SDPTG, and APG.

## SEARCH STRATEGY

The data collected from PubMed database has been used for English literature and ScienceLinksJapan for Japanese literature. The reason behind choosing the PubMed database, it comprises more than 19 million citations for biomedical articles from MEDLINE and life science journals back to 1948.

PubMed includes links to full-text articles which may be found in other databases such as PubMed Central or at publisher web sites.

The total number of references for “Photoplethysmography” terminology was 1024, “Photoplethysmogram” was 91, “PPG”was 232, “PTG”was 13, “DVP” was 10, “APG” was 4, and “SDPTG” was 15 in PUBMED database. 

On the other hand, the total number of references for “Photoplethysmography” terminology was 23, “Photoplethysmogram” was 24, “PPG”was 2, “PTG”was 4, “DVP” was 8, “APG” was 28, and “SDPTG” was 8 in SCIENCELINKS Japanese database.

As shown in Fig. (**2a**); the number of publications for “PPG” is increasing with years more than “PTG” and “DVP”. In Fig. (**2b**), the cumulative sum of PPG clearly shows an exponential increase through years.

As shown in Fig. (**3a**); the number of publications for “APG” is increasing with years more than “SDPTG”. In Fig. (**3b**), the cumulative sum of APG clearly shows an exponential increase through years.

By categorizing the number of publication by its discipline, it is interesting to notice in Fig. (**4a**) that the usage of “PPG “,”PTG”, and “DVP” terminologies is more in Engineering than science “SDPTG”. In Fig. (**4b**), the usage of “APG” and “SDPTG” vanishes in Engineering compared to Science.

The significant result in Fig. (**5**) shows that all Photoplethysmography terminologies are coherent between PubMed and ScienceLinksJapan in all terminologies except APG. APG has been used by Japanese scientist more than western scientist. Therefore, the recommended as standard naming for the discussed terminologies is:

**Table:** 

**Terminology**	**Acronym**
Photoplethysmography Photoplethysmogram Digital Velocity Pulse	PPG
Second Derivative Photoplethysmography Second Derivative Photoplethysmogram Second Derivative Digital Velocity Pulse Acceleration Plethysmography	APG

## CONCLUSION

The author suggests to all scientists and engineers active in photoplethymogram work to use PPG (for photoplethysmogram) and APG (for the second derivative photoplethysmogram) terminologies in oral presentations and written publications. The usage of different terminologies for the same signal can lead to a disconnect between different groups of engineers and scientists. 

These standard terminologies will save the researcher’s efforts and overcome generating redundant results. It will also speed up the scientific progress in this multidisciplinary field. This paper is an attempt to resolve the difficulties and confusion regarding plethysmography terminologies which have persisted for more than 50 years.

## Figures and Tables

**Fig. (1):**
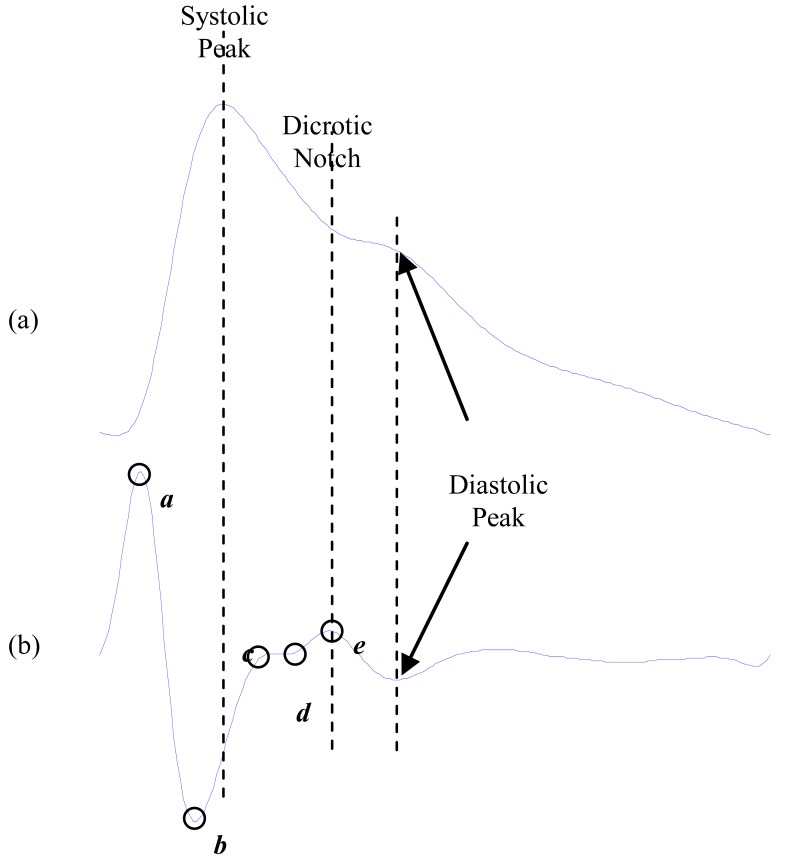
Signal Measurements [5] (**a**) fingertip photoplethysmogram (**b**) second derivative wave of photoplethysmogram. The photoplethysmogram waveform consists of one systolic wave and one diastolic wave while the second derivative photoplethysmogram waveform consists of four systolic waves (*a*, *b*, *c*, and *d* waves) and one diastolic wave (*e* wave).

**Fig. (2):**
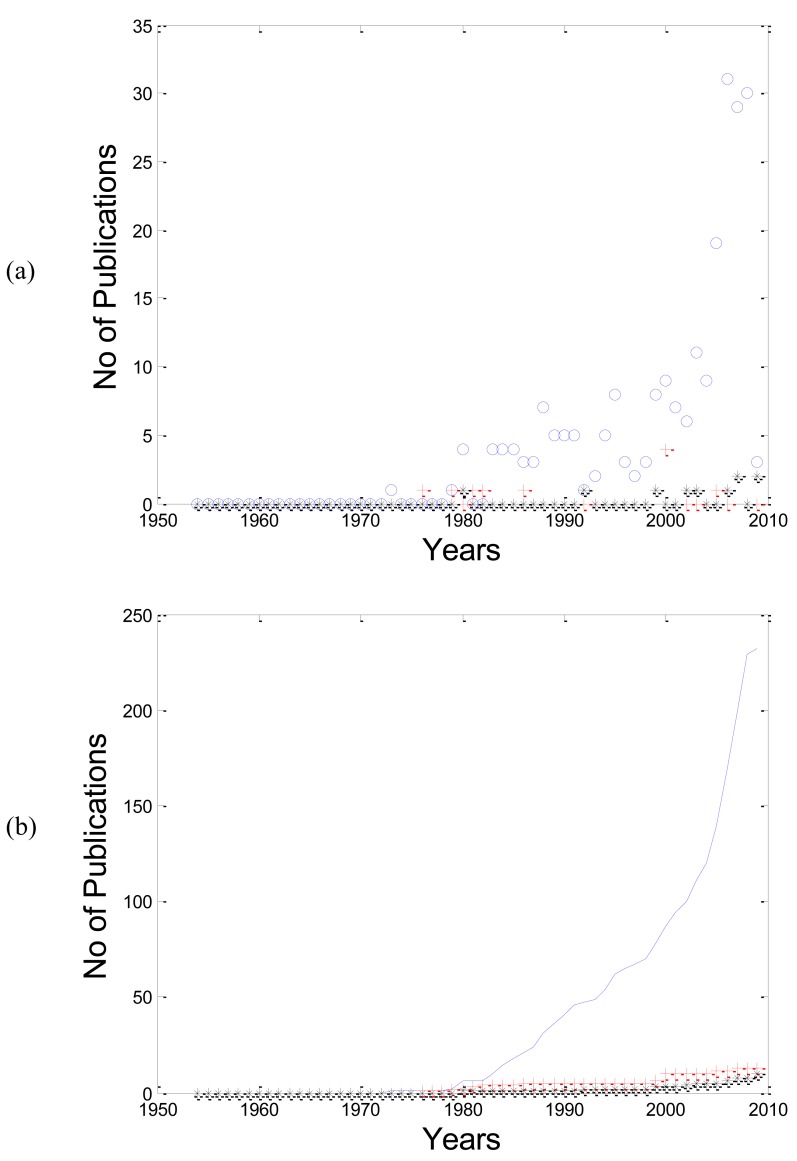
(**a**) the relationship between the photoplethysmogram terminologies (PPG”o”, PTG”+”, and DVP “*”) and the number of publications through years. (**b**) The relationship between the cumulative sum of PPG” solid curve”, PTG “dashed curve”, and DVP” dotted curve” terminologies with their number of publications. It is clear that the use of PPG is increasing exponentially over time.

**Fig. (3):**
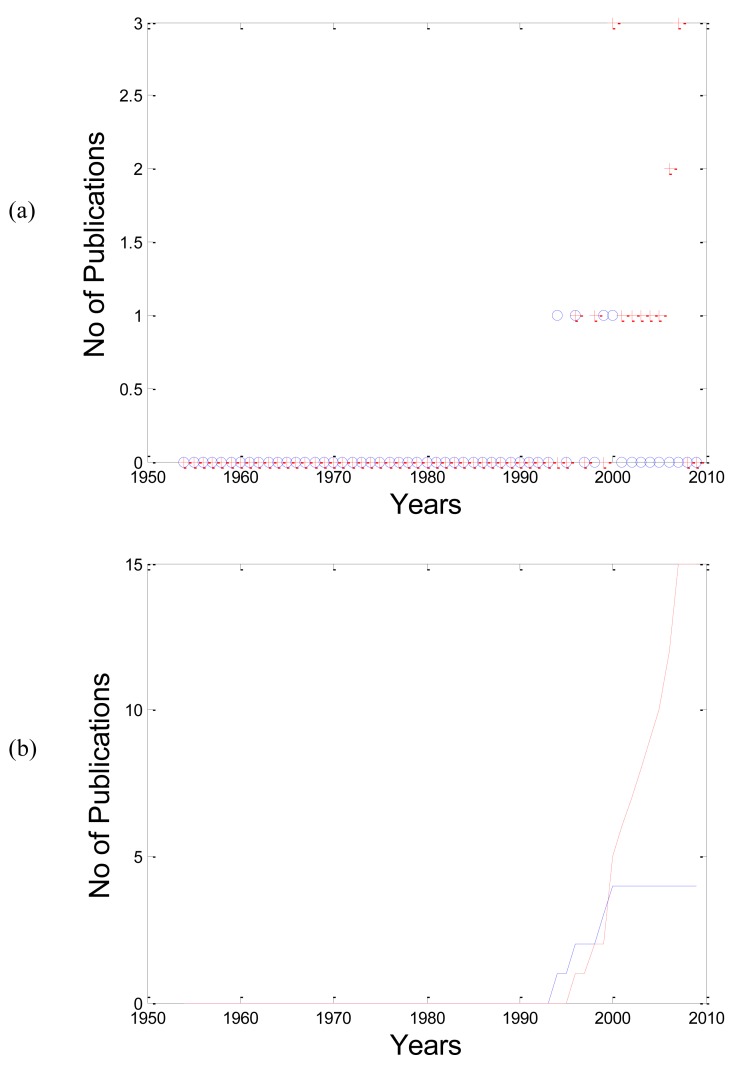
(**a**) The relationship between the acceleration photoplethysmogram terminologies (APG”+” and SDPTG ”o”) and their number of publications through years. (**b**) The relationship between the cumulative sum of APG” solid curve” and SDPTG” dotted curve” terminologies with their number of publications. It is clear that the use of SDPTG is increasing exponentially over time.

**Fig. (4):**
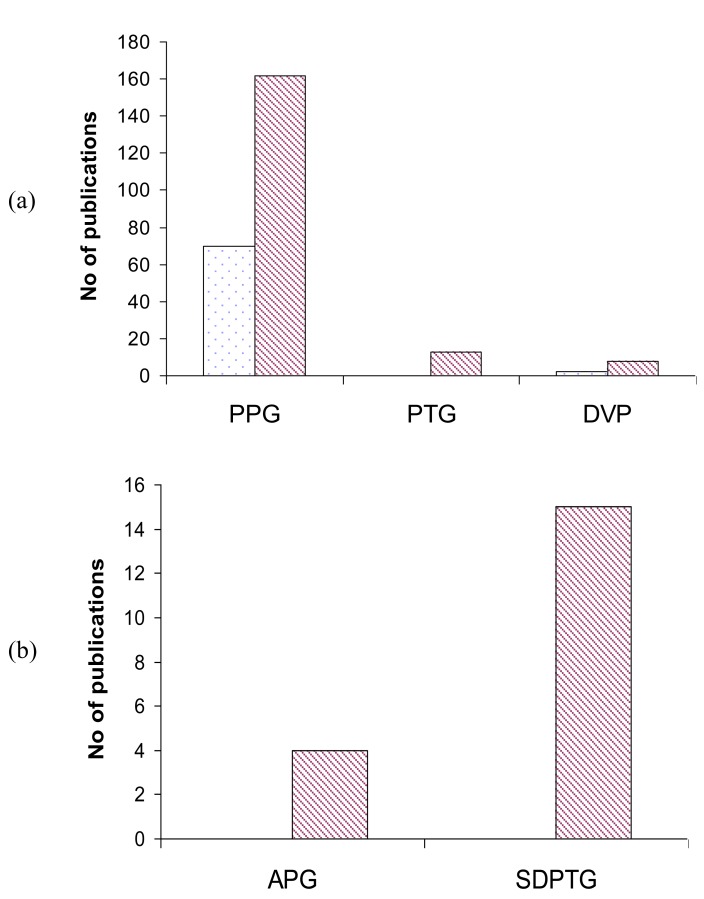
(**a**) usage of PPG, PTG and DVP in engineering field (dotted blocks) and science (dashed blocks). (**b**) usage of APG and SDPTG in science field because the number of publications using APG and SDPTG in engineering field is very limited.

**Fig. (5):**
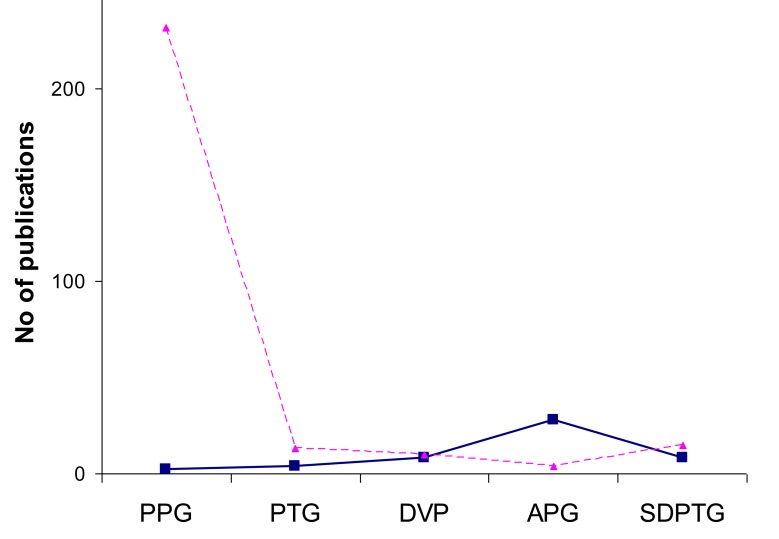
Overall terminologies published in English literature ”dashed line” while the”solid line” in Japanese literature. It is clear that PPG is the most used acronym for photoplethysmogram and APG is the most used acronym for the second derivative of the photoplethysmogram signal.
